# The Methods of Bacteriological Investigation

**Published:** 1886-04

**Authors:** 


**The Methods of Bacteriological Investigation.** By DR. FERDINAND HUEPPE, Docent in Hygiene and Bacteriology in the Chemical Laboratory of R. Fresenius, in Wiesbaden. Translated by HERMANN M. BIGGS, M. D., Instructor in the Carnegie Laboratory, and Assistant to the Chair of Pathological Anatomy in Bellevue Hospital Medical College. Illustrated by thirty-one wood-cuts. New York: D. Appleton & Co., 1, 3 and 5 Bond street. 1886.

Dr. Hueppe is a pupil of Koch, and published this book by the wish of his esteemed teacher. This work, on an important subject, has won for its author deserved praise in Germany, and we trust the translator will be none the less successful. Dr. Biggs has published articles of value on this subject, and has translated the work well, showing a thorough knowledge of the text and theme. To all who desire to study the subject, we recommend the book.

